# Association between Metabolic Syndrome Diagnosis and the Physical Activity—Sedentary Profile of Adolescents with Obesity: A Complementary Analysis of the Beta-JUDO Study

**DOI:** 10.3390/nu14010060

**Published:** 2021-12-24

**Authors:** Valérie Julian, Iris Ciba, Roger Olsson, Marie Dahlbom, Dieter Furthner, Julian Gomahr, Katharina Maruszczak, Katharina Morwald, Thomas Pixner, Anna Schneider, Bruno Pereira, Martine Duclos, Daniel Weghuber, David Thivel, Peter Bergsten, Anders Forslund

**Affiliations:** 1 Department of Sport Medicine and Functional Explorations, Diet and Musculoskeletal Health Team, Human Nutrition Research Center (CRNH), INRA, University Teaching Hospital of Clermont-Ferrand, University of Clermont Auvergne, 63000 Clermont-Ferrand, France; mduclos@chu-clermontferrand.fr; 2 Department of Pediatrics, Paracelsus Medical University, 5020 Salzburg, Austria; j.gomahr@salk.at (J.G.); k.maruszczak@salk.at (K.M.); k.morwald@salk.at (K.M.); an.schneider@salk.at (A.S.); d.weghuber@salk.at (D.W.); 3 Overweight Unit, Children’s Hospital, 752 37 Uppsala, Sweden; iris.ciba@kbh.uu.se (I.C.); marie.dahlbom@kbh.uu.se (M.D.); peter.bergsten@mcb.uu.se (P.B.); 4 Department of Women’s and Children’s Health, Uppsala University, 752 36 Uppsala, Sweden; roger.olsson@kbh.uu.se (R.O.); anders.forslund@kbh.uu.se (A.F.); 5 Department of Pediatrics and Adolescent Medicine, Salzkammergut-Klinikum, 4840 Voecklabruck, Austria; dieterfurthner@hotmail.com (D.F.); tom.pixner@gmx.at (T.P.); 6 Obesity Research Unit, Paracelsus Medical University, 5020 Salzburg, Austria; 7 Department of Biostatistics, University Teaching Hospital of Clermont-Ferrand, 63000 Clermont-Ferrand, France; bpereira@chu-clermontferrand.fr; 8 Laboratory of Metabolic Adaptations to Exercise under Physiological and Pathological Conditions (AME2P), University of Clermont Auvergne, 63000 Clermont-Ferrand, France; david.thivel@uca.fr; 9 Department of Medical Cell Biology, Uppsala University, 752 36 Uppsala, Sweden

**Keywords:** pediatric obesity, metabolic syndrome, sedentary time, physical activity, cardiometabolic risk

## Abstract

Metabolic syndrome (MetS) is highly prevalent in children and adolescents with obesity and places them at an increased risk of cardiovascular-related diseases. However, the associations between objectively measured movement-related behaviors and MetS diagnosis remain unexplored in youths with obesity. The aim was to compare profiles of sedentary (SED) time (more sedentary, SED+ vs. less sedentary, SED−), moderate to vigorous physical activity (MVPA) time (more active, MVPA+ vs. less active, MVPA−) and combinations of behaviors (SED−/MVPA+, SED−/MVPA−, SED+/MVPA+, SED+/MVPA−) regarding the MetS diagnosis. One hundred and thirty-four adolescents with obesity (13.4 *±* 2.2 years) underwent 24 h/7 day accelerometry, waist circumference (WC), blood pressure (BP), high-density lipoprotein-cholesterol (HDL-c), triglycerides (TG) and insulin-resistance (IR) assessments. Cumulative cardiometabolic risk was assessed by using (i) MetS status (usual dichotomic definition) and (ii) cardiometabolic risk z-score (MetScore, mean of standardized WC, BP, IR, TG and inverted HDL-c). SED− vs. SED+ and MVPA+ vs. MVPA− had lower MetS (*p* < 0.01 and *p* < 0.001) and MetScore (*p* < 0.001). SED−/MVPA+ had the lowest risk. While SED and MVPA times were lower in SED−/MVPA− vs. SED+/MVPA+ (*p* < 0.001), MetScore was lower in SED−/MVPA− independently of body mass index (BMI) (*p* < 0.05). MVPA, but not SED, time was independently associated with MetS diagnosis (*p* < 0.05). Both MVPA (*p* < 0.01) and SED times (*p* < 0.05) were associated with MetScore independently of each other. A higher MVPA and lower SED time are associated with lower cumulative cardiometabolic risk.

## 1. Introduction

Approximately 107 million children and adolescents [1] have obesity. This is a major worldwide concern, due to its association with the development of early cardiometabolic comorbidities, such as insulin resistance (IR), dyslipidemia and high blood pressure (BP). These conditions constitute a cluster of chronic cardiometabolic disorders termed metabolic syndrome (MetS) [2]. The MetS prevalence rate is estimated to be up to 30% in adolescents with obesity [3]. Evidence from longitudinal studies shows that pediatric MetS transfers into an increased risk for later cardiovascular diseases and type 2 diabetes mellitus (T2D) [4,5]. The importance of diagnosing the MetS in youths is also driven by its associations with non-alcoholic fatty liver disease (NAFLD), renal dysfunction [6] and premature death [7]. The management of obesity and MetS in children and adolescents is currently based on multidisciplinary interventions combining nutritional guidelines and physical activity (PA), particularly moderate-to-vigorous PA (MVPA) [8,9,10]. Daily MVPA is negatively associated with body mass index (BMI), adiposity, waist circumference (WC), obesity-related cardiometabolic comorbidities (i.e., IR, dyslipidemia and high BP) and the MetS diagnosis [11,12,13,14,15,16,17,18].

However, one can be active (i.e., high MVPA) while also being involved in high amounts of daily sedentary (SED) behaviors (i.e., waking behavior in a sitting, reclining or lying posture) [19]. Strong evidence in adults showed that PA reduced without eliminating the cardiometabolic risk associated with high SED time [20]. In youths, recent studies demonstrated that SED time was positively associated with BMI, adiposity, WC, obesity-related cardiometabolic comorbidities and the MetS diagnosis [18,19,21,22,23,24]. Thus, behavioral pediatric recommendations, which mainly focused on MVPA so far, also advocate now, in addition to MVPA guidelines, for the minimization of SED time [25]. To our knowledge, the associations between objectively and comprehensively measured movement-related behaviors (i.e., both MVPA and SED times) and the METS diagnosis remain unexplored in youths with obesity. Interestingly, in a recent cross-sectional study conducted on middle-aged individuals of similar BMI (with and without obesity), Bowden-Davies et al. (2019) showed that subjects with MetS diagnosis were more sedentary compared to subjects without MetS, and that overall PA did not account for differencing metabolic health status. These results thus suggest that treatment strategies might widely benefit from reducing SED time [26]. 

Because it remains difficult to implement MVPA in youths with obesity, decreasing SED time could be explored as a more feasible first step to decrease MetS in clinical practice. Our team recently conducted a cross-sectional analysis comparing 7 day/24 h device-based measured profiles of SED time (more sedentary, SED + vs. less sedentary, SED−), MVPA time (more active, MVPA+ vs. less active, MVPA−) and combinations of SED and MVPA times (SED−/MVPA+, SED−/MVPA−, SED+/MVPA+, SED+/MVPA−) in regard to continuous/single parameters (body composition, IR, lipid profile and BP parameters), without considering the MetS diagnosis, in children and adolescents with obesity. Since analyzing combined profiles of SED and MVPA times was relevant in regard to continuous/single parameters, it seems important to conduct a secondary analysis based on the same population but focusing on the associations between MVPA and SED times profiles and the MetS diagnosis. 

Therefore, the aim of the present study was to compare profiles of SED time, MVPA time and combinations of SED time and MVPA times in regard to the cumulative cardiometabolic risk, as assessed by (i) the MetS status (i.e., the usual clinical dichotomic definition of the MetS, using strict thresholds) and (ii) a computed continuous z-score of clustered cardiometabolic risk (i.e., MetScore, calculated from the same criteria than those using to diagnose the MetS). The present study also investigated the correlations between SED and different intensities levels of PA times and the MetS diagnosis. 

## 2. Materials and Methods

### 2.1. Subjects

The study involved 134 children and adolescents with obesity, aged 10 to 17 years-old (referred to as “adolescents” hereafter), participating in the “Beta-cell function in Juvenile Diabetes and Obesity” (Beta-JUDO) study. It is a large translational project that aims to define the mechanisms of and how to counteract insulin hypersecretion in children and adolescents with obesity. The entire project and the primary publications’ references are available on the CORDIS website [27]. More recently, the Beta-JUDO study consortium also investigated the underlying mechanisms of disturbed glucose metabolism and non-alcoholic fatty liver disease [28,29,30,31,32,33]. The subjects were included in 2 centers (Pediatric Obesity Clinic at Uppsala University Children’s Hospital, Upsala, Sweden; and Paracelsus Medical University, Salzburg, Austria). Obesity was defined as age-adapted BMI > 30 kg/m^2^. The study was accepted for Voluntary Harmonisation Procedure (VHP673, VHP2015061) and approved by Ethics Committees and Regulatory Authorities in Sweden and Austria (EudraCT No. 2015-001628-45; EC Sweden, Dnr 2015/279; EC Austria, 415-E/1544/20-2014). Written informed consent was obtained from participants and parents. The trial was conducted according to the Declaration of Helsinki (World Medical Association; Version 2013) and the E6 Guideline for Good Clinical Practice (International Conference on Harmonisation). 

### 2.2. Anthropometry

All the measurements were performed by using standard operating procedures harmonized between centers [33]. Weight (kg) was measured with a standardized calibrated scale (Uppsala: SECA model 704; Salzburg: SECA model 801, Hamburg, Germany). Height (cm) was assessed by using a stadiometer (Uppsala: Ulmer stadiometer, Busse, Elchingen, Germany; Salzburg: SECA, model 222 stadiometer, Hamburg, Germany). BMI was calculated as weight in kilograms divided by the square of height in meters. The BMI-SDS (Microsoft Excel add-in LMS Growth, using WHO growth report Version 2.76) and the BMI in percentiles (WHO BMI for age) were calculated. Waist circumference (WC, cm) was measured on the standing subject with a flexible tape midway between the superior border of the iliac crest and the lowest rib.

### 2.3. Blood Pressure

Systolic and diastolic blood pressure were measured in a sitting position on the right arm, after 5 min of quiet rest (mean of two measurements), with a standardized clinical aneroid sphygmomanometer and appropriate cuffs (Uppsala: CAS 740, CAS Medical Systems, Inc., Branford, CT, USA; Salzburg: Carescape V100, Dinamap Technology/GE, Vienna, Austria).

### 2.4. Blood Sampling

Fasting-blood samples were analyzed locally at Uppsala and Salzburg hospitals. Validation of analyses was performed between the two laboratories, using reference blood samples. Total cholesterol, high-density lipoprotein cholesterol (HDL-c), low-density lipoprotein cholesterol (LDL-c) and triglycerides (TG) were analyzed by enzymatic photometric analysis. Plasma glucose was analyzed by enzymatic chromatic test. Plasma was used for central analyses of insulin, using singleplex enzyme-linked immunosorbent assay kits for each analyte (Mercodia AB, Uppsala, Sweden). The homeostasis model assessment of insulin-resistance index (HOMA-IR) was calculated by using the following formula: [HOMA-IR] = glycemia [mmol⋅L^−1^] × insulinemia [mUI⋅L^−1^]/22.5) [34]. 

### 2.5. Metabolic Syndrome Diagnosis

MetS was diagnosed according to a usual clinical dichotomic definition adapted from Chen and collaborators [35] and previously used in similar populations [36,37], considering the presence of three or more of the following criteria: WC ≥ 97th percentiles for age and sex; SBP or DBP ≥ 90th percentile; HDL-c ≤ 0.4 g·L^−1^, TG ≥ 1.3 g·L^−1^ and HOMA-IR > 75th percentile. 

### 2.6. Continuous Cardiometabolic Syndrome Score 

MetScore was computed by following the method previously used for pediatric populations without [38] or with obesity [37]. Z-scores were calculated for the following variables (same criteria as above): WC, mean BP, HDL-c, TG and HOMA-IR. The scores were computed by subtracting the sample mean from the subject mean and then dividing by the standard deviation (SD), as follows: Z = ([value − mean]/SD. The mean of this score is therefore zero. The scores were multiplied by −1 for HDL-c. Higher values thus indicate higher cardiometabolic risk. The total MetScore was calculated by the sum of Z-scores for each variable divided by 5. This continuous score is relevant to take into account the degree of development of each of the 5 components of the MetS (and not only components that would remain under the cut points) [37]. 

### 2.7. Physical Activity and Sedentary Time 

Physical activity was assessed by registration with the accelerometer Actical^®^ (Philips Respironics, Inc., Murrysville, PA, USA), which is an omni-directional waterproof device. It records accelerations in the range of 0.05–2.0 g and is sensitive to movements in the range of 0.35–3.5 Hz. The Actical^®^ monitor has an internal time clock and extended memory and is able to record the magnitude of acceleration and deceleration associated with every movement. The recorded signal is scored as a “count” which is summed over a 1-min epoch. Participants were given the accelerometer at the examination center and were asked to wear the Actical^®^ device on their non-dominant wrist during 7 consecutive days and nights (24-h measurements). Non-wear time was defined as ≥60 consecutive minutes of zero counts, with allowance for 2 min of counts between zero and 100. To be included in the analyses, participants were required to have 5 of 7 valid days with at least one of the valid day on a weekend. Each minute of wear time was classified by using established cut points into SED (<1.5 metabolic equivalent of the task), light PA (LPA, <3 metabolic equivalent of the task), moderate PA (MPA, 3 to 6 metabolic equivalent of the task) and vigorous PA (VPA, superior to 6 metabolic equivalent of the task) [33,39]. MVPA was calculated as the sum of MPA and VPA. SED+ (more sedentary) and SED− (less sedentary) groups were defined by being respectively above and under the median of the sample for SB time. MVPA+ (more active) and MVPA− (less active) groups were defined by being respectively above and under the median of the sample for MVPA time. The 4 combinations of movement-related behaviors (SED-/MVPA+, SED−/MVPA−, SED+/MVPA+, SED+/MVPA−) were created by using the median of MVPA for each of the SED subsamples.

### 2.8. Statistics

Continuous data were expressed as mean and standard deviation. The assumption of normality distribution was checked with the Shapiro–Wilk test and histograms graphs. The following variables did not meet the assumption of normality: SED time, LPA time, MPA time, VPA time, MVPA time, fasting glucose, fasting insulin, HOMA-IR, TG, LDL-c, total-c, TG score and IR score. The comparisons between groups (SED+ and SED−, MVPA+ and MVPA− and SED−/MVPA+, SED−/MVPA−, SED+/MVPA+ and SED+/MVPA−) were performed by using Chi-squared or Fisher’s exact tests for categorical data, whereas the comparisons for continuous variables were conducted by using analysis of variance (ANOVA) or non-parametric Kruskal–Wallis test, when the assumptions of ANOVA were not met. The homoscedasticity was studied by using Bartlett’s test. Multivariable analyses were then conducted by using multiple regression (linear for continuous dependent variable and logistic for binary) in order to compare groups adjusting aforementioned analyses on possible confounders. The covariates were chosen with caution according to the univariate results and to clinical relevance. The testing and parameter estimation performed by using a statistical model clearly depend on the variables included in the model. It is therefore crucial for confounding adjustment that known clinically significant variables are included in the regression model. The multivariable regression analyses were run with the following adjustment covariates: age, gender, Tanner stages and BMI. The normality of residuals was checked for multiple linear models. When appropriate, a logarithmic transformation of the dependent variable was performed. The relationships between continuous data were explored by estimating Pearson or Spearman correlation coefficient, with a Sidak type I error correction to take into account multiple comparisons. A heatmap graph was used to describe these correlations. Statistical analyses were performed by using Stata software (version 15, StataCorp, College Station, TX, USA). Differences were considered statistically significant at *p* < 0.05. Venn diagrams were used to represent the proportions of subjects meeting each criterion of MetS for each group.

## 3. Results

One hundred and thirty-four adolescents (mean age 13.4 ± 2.2 years, 48.5% females) were included (*n* = 119 from Uppsala and *n* = 15 from Salzburg). The mean BMI was 98.9 ± 0.7 kg⋅m^−2^. Sixty-three percent (*n* = 85) had a MetS. WC criteria were met by all individuals of the present study. Mean accelerometry wear time was 6.5 ± 1.1 days, with 99.4 ± 2.9 percent of daily wear time. Anthropometric, accelerometry, cardiometabolic variables and MetS status for overall sample are presented in Table 1.

Anthropometric, accelerometry and single/continuous cardiometabolic variables for SED−, SED+, MVPA−, MVPA+, SED−/MVPA+, SED−/MVPA−, SED+/MVPA+ and SED+/MVPA− groups are presented in Supplementary Materials Table S1.

### 3.1. Comparison SED+ vs. SED− Groups

**Figure 1:**
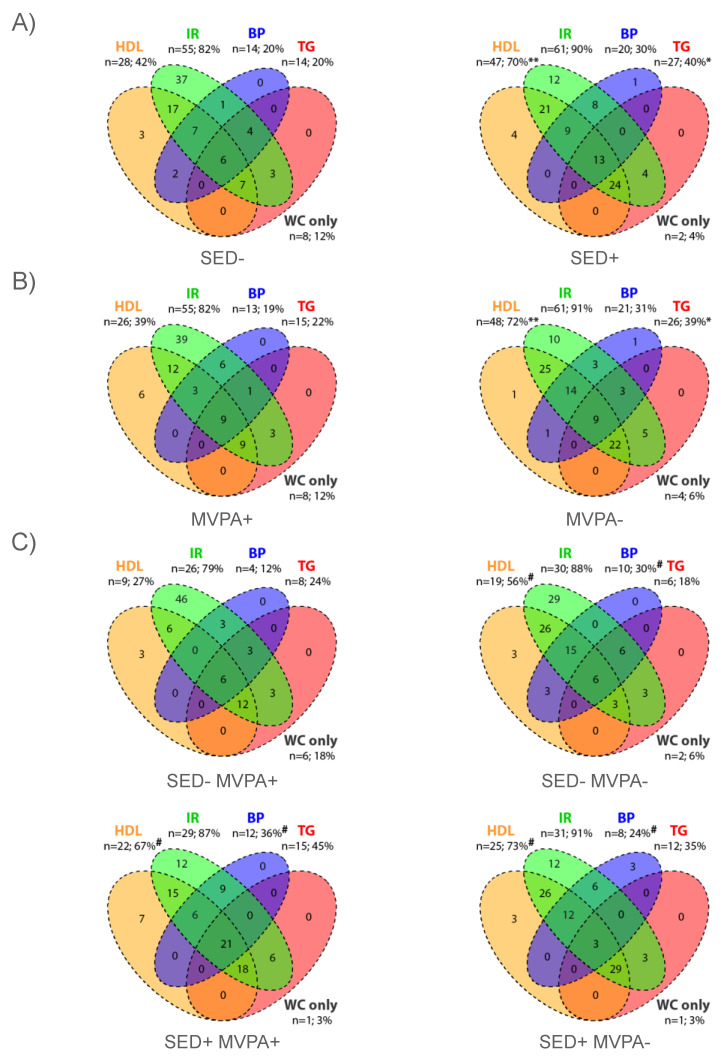
Venn diagram showing proportions (%) of subjects meeting each criterion of MetS for (**A**) SED− (*n* = 67) and SED+ (*n* = 67) groups; (**B**) MVPA+ (*n* = 67) and MVPA− (*n* = 67) groups; and (**C**) SED−/MVPA+ (*n* = 33), SED−/MVPA− (*n* = 34), SED+/MVPA+ (*n* = 33) and SED+/MVPA− (*n* = 34) groups. The orange circle represents subjects with low high-density lipoprotein (HDL) cholesterol. The green circle represents subjects with high insulin resistance (IR). The purple circle represents subjects with high blood pressure (PB). The red circle represents subjects with hypertriglyceridemia (TG). BP, blood pressure; IR, insulin resistance; HDL, high-density lipoprotein; TG, triglycerides. Different between SED+ vs. SED− groups and between MVPA− vs. MVPA+ groups: * *p* < 0.05, ** *p* < 0.01. Different from SED−/MVPA+: ^#^ *p* < 0.05.

The SED+ group had a higher SED time, but lower total PA, MVPA and LPA times in comparison to SED− group (*p* < 0.001) (Table S1). The number of subjects presenting a MetS (*p* < 0.01), the total MetScore (*p* < 0.001) and the mean number of MetS components (*p* < 0.001) were higher in the SED+ group in comparison to the SED− group when adjusted for age, gender and Tanner stages (Table 2), which remained significant after adjustment with age, gender, Tanner stages and BMI (*p* < 0.05). The proportions of subjects meeting each criterion of the MetS for the SED− and SED+ groups are presented in Figure 1A. The numbers of subjects presenting HDL and TG criteria were higher in the MVPA− group in comparison to the MVPA− group (*p* < 0.05). 

### 3.2. Comparison MVPA− vs. MVPA+ Groups

The MVPA− group had a lower total PA time, a lower MVPA time and a higher SED time in comparison to MVPA+ group (*p* < 0.001) (Supplementary Materials Table S1). The number of subjects presenting a MetS (*p* < 0.001), the total MetScore (*p* < 0.001) and the mean number of MetS components (*p* < 0.001) were higher in MVPA− group in comparison to MVPA+ group when adjusted for age, gender and Tanner stages (Table 2), which remained significant after adjustment with age, gender, Tanner stages and BMI (*p* < 0.05). The proportions of subjects meeting each criterion of the MetS for MVPA- and MVPA+ groups are presented in Figure 1B. The numbers of subjects presenting HDL and TG criteria were higher in the MVPA− group in comparison to the MVPA- group (*p* < 0.05).

### 3.3. Comparison between SED−/MVPA+, SED−/MVPA−, SED+/MVPA+ and SED+/MVPA− Groups

The SED-/MVPA+ group had a higher MVPA time in comparison to the SED−/MVPA−, SED+/MVPA+ and SED+/MVPA− groups (*p* < 0.001) and a lower SED time in comparison to SED+/MVPA+ and SED+/MVPA− (*p* < 0.001) (Supplementary Materials Table S1). The number of subjects presenting a MetS and the mean number of MetS components were lower in the SED−/MVPA+ group compared to the other groups (*p* < 0.05) when adjusted for age, gender and Tanner stages. The total MetScore was lower in the SED−/MVPA+ group compared to the SED+/MVPA− group and the SED+/MVPA+ group (*p* < 0.001) when adjusted for age, gender and Tanner stages (Table 2). After adjustment with age, gender, Tanner and BMI, the differences concerning the number of subjects presenting a MetS and the mean number of MetS components were no longer significant (*p* > 0.05) but the total MetScore remained lower in the SED−/MVPA+ group compared to the SED+/MVPA− group (*p* < 0.05). The proportions of subjects meeting each criterion of MetS in the four groups are presented in Figure 1C. The number of subjects presenting HDL and BP criteria were lower in the SED−/MVPA+ group compared to the other groups (*p* < 0.05). 

Interestingly, the SED−/MVPA− group had a lower SED time (*p* < 0.001), a lower MVPA time (*p* < 0.001) and a higher LPA time (*p* < 0.001) in comparison with the SED+/MVPA+ group. BMI was not significantly different between the two groups (*p* > 0.05) (Supplementary Materials Table S1). Neither the number of subjects presenting a MetS nor the mean number of MetS components were significant between the two groups (*p* > 0.05). However, the MetScore was higher in the SED−/MVPA− group compared to the SED+/MVPA+ group when adjusted for age, gender and Tanner stages (*p* < 0.01) (Table 2), which remained significant after adjustment with age, gender, Tanner stages and BMI (*p* < 0.05).

### 3.4. Correlations

**Figure 2:**
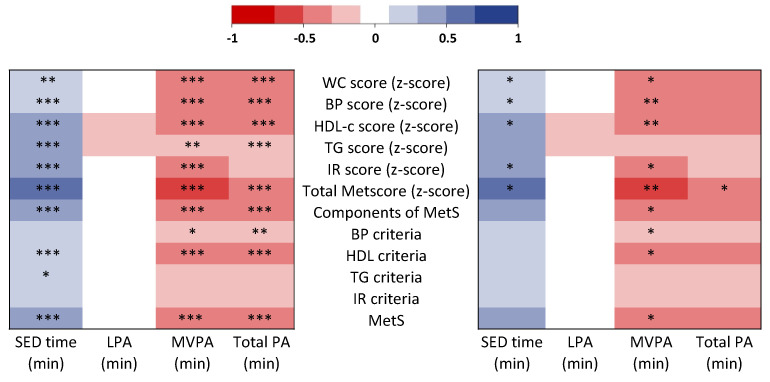
Heatmap representation of the correlations between accelerometry variables, MetS status, MetS criteria and continuous z-scores of cardiometabolic risks (MetScore). The darker the color (blue or red) of the box is, the higher the value (negative for red; positive for blue) of the correlation. Left: unadjusted model. Right: adjusted for sedentary time when physical was modeled as the main exposure and adjusted for MVPA when sedentary time was modeled as the main exposure. * *p*-value < 0.05, ** *p*-value < 0.01 and *** *p*-value < 0.001. BP, blood pressure; HDL-c, high-density lipoprotein-cholesterol; IR, insulin resistance; LPA, light physical activity; MetS, metabolic syndrome; MVPA, moderate-to-vigorous physical activity; SED, sedentary; TG, triglycerides; WC, waist circumference.

All correlations between accelerometry variables, MetS status, MetS criteria and continuous z-scores of cardiometabolic risks are presented in Figure 2. SED time was positively correlated with the MetS status (*p* < 0.001), the total MetScore (*p* < 0.001) and the mean number of MetS components (*p* < 0.001). After adjustment with MVPA time, the correlation between SED time and total MetScore remained significant (*p* < 0.05), which was not the case for the associations between SED time and the MetS status or the mean number of MetS components (*p* > 0.05). SED time was positively correlated with WC score (*p* < 0.01), BP score (*p* < 0.001), HDL-c score (*p* < 0.001), IR score (*p* < 0.001) and TG score (*p* < 0.001), which remain significant after adjustment with SED time (*p* < 0.05), except for the TG score.

The MVPA time was negatively correlated with the MetS status (*p* < 0.001), the total MetScore (*p* < 0.001) and the mean number of MetS components (*p* < 0.001). These correlations remained significant after adjustment with SED time (*p* < 0.05, *p* < 0.01 and *p* < 0.05, respectively). MVPA was negatively correlated with WC score (*p* < 0.001), BP score (*p* < 0.001), HDL-c score (*p* < 0.001), IR score (*p* < 0.001) and TG score (*p* < 0.01), which remain significant after adjustment with SED time (*p* < 0.01 for BP and HDL-c scores, *p* < 0.05 for C and IR scores), except for TG score. 

The total PA time was negatively correlated with the MetS status (*p* < 0.001), the total MetScore (*p* < 0.001) and the mean number of MetS components (*p* < 0.001). The correlation between total PA time and total MetScore remained significant after adjustment with SED time (*p* < 0.05), but this was not the case for the associations between total PA time and the MetS status nor the mean number of MetS components (*p* > 0.05). 

LPA was not significantly associated with the MetS status, the total MetScore or the mean number of MetS components (*p* > 0.05).

## 4. Discussion

This study aimed to investigate the associations between SED and MVPA profiles objectively assessed by 24 h/7 days accelerometry and the cumulative cardiometabolic risk, as measured by the MetS status (i.e., diagnosis of MetS based on the usual clinical definition using dichotomic criteria) and a computed continuous z-score of clustered cardiometabolic risks (based on the same criteria than the dichotomic definition), in adolescents with obesity. According to our analysis, SED− and MVPA+ groups have a lower cumulative cardiometabolic risk than SED+ and MVPA− groups, respectively, independent of age, gender, maturation and BMI. As expected, the SED−/MVPA+ profile had the lowest cumulative cardiometabolic risk. However, as the SED−/MVPA− profile had a lower continuous z-score of cardiometabolic risks in comparison with the SED+/MVPA+ profile, independent of BMI, reducing SED time might be beneficial whatever the level of MVPA. Furthermore, both the MetS status and the continuous z-score of cardiometabolic risks were positively correlated with SED time and negatively correlated with MVPA time. 

The population of the present study is comparable with previously studied populations of youths with similar BMI in regard to the prevalence of the MetS (63%) and cardiometabolic risks criteria [40,41]. As previously described, IR was the MetS criterion with the highest prevalence [37]. 

The present results are fully concordant with studies demonstrating that both sedentary and MVPA times would positively impact adiposity [23], cardiometabolic risk factors [19,21,22] and the MetS status [18] in youths. The lower cumulative cardiometabolic risk found in the SED−/MVPA+ profile is in keeping with recent studies showing that youths meeting both MVPA and SED recommendations are less likely to develop obesity and to present related cardiometabolic risks [42,43,44]. Moreover, the lower continuous z-score of cardiometabolic risks found in the SED−/MVPA− group in comparison to the SED+/MVPA+ group strengthens previous findings observed in adults with and without obesity, showing a negative impact of SED behaviors on health outcomes, even when concurrent levels of MVPA are considered [26,45]. 

The results of the presented mutually adjusted model of correlations between movement-related behaviors and the dichotomous diagnosis of the MetS are also in line with a recent meta-analysis showing that PA of at least moderate intensity, but not SED time, was independently associated with the presence of the MetS defined with dichotomous criteria in children and adolescents [18]. For Fridolfsson et al., the association between SED time and cardiometabolic health is generally weaker in contrast to high-intensity PA in youths [46]. However, the independence of the associations between movement-related behaviors and obesity-related health parameters remains debatable [18,23,46,47,48], as some recent longitudinal studies have reported significant positive associations between SED behaviors, BMI and fat mass index during childhood and adolescence, even after adjustment for MVPA [47,48]. In the present study, the independence of the association between SED time and the continuous z-score of cardiometabolic risks is in line with these last results [47,48] and highlights the importance of reducing SED time in order to improve cardiometabolic health in adolescents with obesity, whatever the level of MVPA. Taken together, the present findings indicate that in adolescents with obesity, reducing SED time is needed (in addition to the increase of MVPA). As there are many barriers in clinical practice leading to MVPA intolerance in youths with obesity (e.g., impaired cardiorespiratory fitness and strength related to body weight, restrictions for weight-bearing activities, musculoskeletal pain and discomfort during and after exercising), particularly in early cares, reducing SED time could be a first step toward increased MVPA in order to decrease the cumulative cardiometabolic risk (when increasing MVPA in the same time is not possible). The present results thus strengthen recent obesity treatment strategies consisting in breaking up extended periods of SED time, replacing it with MVPA (the preferred and more efficient scenario) or with LPA (beneficial but with lower effect estimates). These strategies would give the chance to be more flexible by acting on different combinations of time spent in MVPA, LPA and sedentary behaviors [49,50,51,52]. In clinical practice, this means that youths with obesity should benefit from an individual behavioral diagnostic, targeting different intensities of PA and SED behaviors, and that stakeholders need to be sensitized to support the shift from long periods of SED time to daily routines incorporating bouts of PA. Recreational screen time, which would have strong associations with adverse health outcomes [22], could be specifically targeted. 

Our findings have to be considered in light of some limitations. First, the relative small sample size may partially explain lack of significances (e.g., between MVPA− vs. MVPA+ group for BP z-score and between SED+ vs. SED− group for TG z-score). Second, due to its cross-sectional design, the observed associations cannot be interpreted to reflect causal relationships. Moreover, the dichotomic nature of the MetS could explain some lacks of significance, as it does not take into account components that are close to cut points but remain below them [37,38]. Of note, the continuous z-score of cardiometabolic risks (computed in the present study using the same criteria as used to define the METS diagnosis) indeed reflects more precisely the degree of development of each cardiometabolic risk [37] and is known to be statistically more sensitive and less error prone by comparison to dichotomous approaches with strict thresholds [53]. Finally, while the present population is comparable to previously studied populations of adolescents concerning SED time, MVPA time may be overestimated (at the expense of LPA) [54]. It has been shown that Actical is comparable to the gold standard Actigraph GT3X [55], but the time spent in MVPA largely differs across attachment sites (wrist vs. hip) and cut-points in adolescents with obesity [56,57]. The wrist location has been chosen to increase wear compliance [57,58], which is a strength of the present study, denoting measurements with more than 99% of wear time during a mean of 6.5 days. Nevertheless, wrist worn accelerometers can provide higher values of MVPA than hip-worn accelerometers [56,57,58]. However, it is worth noting the major geographical differences in PA patterns, with Swedish children (representing more than two third of the present sample) having a meaningful higher MVPA than other European countries [46].

## 5. Conclusions

In adolescents with obesity, a low sedentary/high MVPA profile is most desirable in regard to the risk for MetS. The lower continuous score of cardiometabolic risks found in the SED−/MVPA− profile in comparison with the SED+/MVPA+ profile suggests that reducing SED behaviors might be beneficial, irrespectively of MVPA level. While MVPA, but not SED time, seems to be independently associated with the MetS diagnosis (based on dichotomous criteria and strict thresholds), both MVPA and SED times are associated with the continuous z-score of cardiometabolic risks, independent of each other. In clinical practice, this means that reducing SED time might be considered as a first step in order to decrease the cumulative cardiometabolic risk in youths with obesity when implementing MVPA is not possible. Stepwise interventional studies, including a first phase of effective reduction of sedentary time preceding a classic MVPA training, would be needed. 

## Figures and Tables

**Table 1:** Anthropometric, accelerometry, cardiometabolic variables and the metabolic syndrome (MetS) status for overall sample (*n* = 134).

**Anthropometry variables**	**Mean ± SD**
Age (year)	13.4 ± 2.2
Females (*n*, %)	65 (48.5)
Tanner stage	3.8 ± 1.3
Height (cm)	164.1 ± 13.6
Weight (kg)	93.2 ± 25.1
BMI (kg⋅m^−2^)	34.3 ± 5.2
SDS-BMI (z-score)	3.18 ± 0.48
BMI (percentile)	98.9 ± 0.7
WC (cm)	109.0 ± 13.8
**Accelerometry variables**	**Mean ± SD**
Sedentary time (min⋅day^−1^)	640 ± 116
LPA (min⋅day^−1^)	484 ± 107
MPA (min⋅day^−1^)	186 ± 76
VPA (min⋅day^−1^)	6 ± 10
MVPA (min⋅day^−1^)	192 ± 81
Total PA (min⋅day^−1^)	676 ± 139
**Cardiometabolic variables**	**Mean ± SD**
Systolic BP (mmHg)	119 ± 12
Diastolic BP (mmHg)	72 ± 9
HDL-cholesterol (mmol⋅L^−1^)	1.06 ± 0.23
Triglycerides (mmol⋅L^−1^)	1.22 ± 0.58
LDL-cholesterol (mmol⋅L^−1^)	2.61 ± 0.81
Total cholesterol (mmol⋅L^−1^)	3.92 ± 0.90
Fast glucose (mmol⋅L^−1^)	5.55 ± 0.59
Fast insulin (mUI⋅L^−1^)	24.12 ± 14.2
HOMA-IR	6.02 ± 3.91
**MetS status and components**	
Components of MetS (mean ± SD)	2.73 ± 0.86
METS (≥3 criteria) (*n*, %)	85 (63)
BP criteria (*n*, %)	34 (25)
HDL criteria (*n*, %)	75 (56)
TG criteria (*n*, %)	41 (31)
IR criteria (*n*, %)	116 (87)

BMI, body mass index; BP, blood pressure; HDL, high-density lipoprotein; HOMA-IR, homeostasis model assessment of insulin resistance; IR, insulin resistance; LDL, low-density lipoprotein; LPA, light physical activity; MetS, metabolic syndrome; MPA, moderate physical activity; MVPA, moderate to vigorous physical activity; PA, physical activity; TG, triglycerides; VPA, vigorous physical activity.

**Table 2:** Metabolic syndrome (MetS) status, mean number of MetS components and continuous z-scores of cardiometabolic risks for SED−, SED+, MVPA+, MVPA−, SED−/MVPA+, SED−/MVPA−, SED+/MVPA+ and SED+/MVPA− groups (mean ± SD or *n*, %).

	SED− *n* = 67	SED+ *n* = 67	MVPA+ *n* = 67	MVPA− *n* = 67	SED−/MVPA+ *n* = 33	SED−/MVPA− *n* = 34	SED+ /MVPA+ *n* = 33	SED+ /MVPA− *n* = 34
Components of MetS	2.67 ± 1.10	3.29 ± 1.04 ***	2.62 ± 1.13	3.34 ± 0.97 ***	2.42 ± 1.14	2.91 ± 1.02 ^#^	3.23 ± 0.89 ^#^	3.36 ± 1.19 ^###^
MetS (≥3 criteria) (*n*, %)	33 (49)	52 (77) **	29 (43)	56 (83) ***	11 (33)	22 (65) ^#^	25 (75) ^#^	27 (79) ^#^
WC score (Z-score)	−0.29 ± 1.05	0.29 ± 0.84 *	−0.35 ± 1.04	0.35 ± 0.81 **	−0.68 ± 0.88	0.07 ± 1.08 ^#^	0.24 ± 0.90 ^#^	0.35 ± 0.79 ^#^
BP score (Z-score)	−0.26 ± 0.90	0.26 ± 1.03 *	−0.25 ± 0.91	0.25 ± 1.01	−0.41 ± 0.88	−0.09 ± 0.88	0.18 ± 0.89 ^#^	0.31 ± 0.92 ^##^
HDL score (Z-score)	−0.40 ± 1.00	0.40 ± 0.82 **	−0.34 ± 1.02	0.34 ± 0.85 **	−0.70 ± 1.01	−0.13 ± 0.91 ^#^	0.36 ± 0.78 ^###,$^	0.45 ± 0.85 ^###,$^
TG score (Z-score)	−0.23 ± 0.96	0.23 ± 0.99	−0.27 ± 0.89	0.27 ± 1.02 *	−0.24 ± 1.01	−0.21 ± 0.91	0.20 ± 0.93	0.25 ± 1.05
IR score (Z-score)	−0.37 ± 0.75	0.37 ± 1.08 ***	−0.27 ± 0.95	0.27 ± 0.97 **	−0.46 ± 0.68	−0.28 ± 0.80	0.36 ± 1.20 ^##,$^	0.38 ± 0.96 ^###,$^
Total MetScore (Z-score)	−0.31 ± 0.57	0.31 ± 0.56 ***	−0.30 ± 0.64	0.30 ± 0.49 ***	−0.50 ± 0.54	−0.13 ± 0.55	0.27 ± 0.53 ^###,$$^	0.35 ± 0.48 ^###,$$^

BP, blood pressure; HDL, high-density lipoprotein; IR, insulin resistance; MetS, metabolic syndrome; TG, triglycerides. Different between SED+ vs. SED− groups and between MVPA− vs. MVPA+ groups: * *p* < 0.05, ** *p* < 0.01, *** *p* < 0.001. Different from SED-MVPA+: ^#^ *p* < 0.05; ^##^ *p* < 0.01; ^###^ *p* < 0.001. Different from SED−MVPA−: ^$^ *p* < 0.05; ^$$^ *p* < 0.01. The *p*-values were adjusted with age, gender and Tanner.

## Supplementary Materials

The following are available online at https://www.mdpi.com/article/10.3390/nu14010060/s1. Table S1: Anthropometric, accelerometry and continuous/single cardiometabolic variables for SED−, SED+, MVPA+, MVPA−, SED−/MVPA+, SED−/PA−, SED+/MVPA+ and SED+/MVPA− (Mean ± SD). *p*-Values are adjusted with age, gender and Tanner.
