# Supplementary material for: Association between Metabolic Syndrome Diagnosis and the Physical Activity—Sedentary Profile of Adolescents with Obesity: A Complementary Analysis of the Beta-JUDO Study
Source: Nutrients. 2021 Dec 24;14(1):60. doi: 10.3390/nu14010060 (PMC8746544; doi:10.3390/nu14010060)
Supplement: Supplementary file 1 [file nutrients-14-00060-s001.zip › nutrients-1495844-supplementary.pdf]

Table S1: Anthropometric, accelerometry and continuous/single cardiometabolic variables for SED-, SED**+**, MVPA**+**, MVPA-, SED-/MVPA**+**, SED-/PA-, SED**+/**MVPA**+** and SED**+/**MVPA- (Mean ± SD). P-values are adjusted with age, gender and Tanner.

	**SED-** n = 67	**SED+** n = 67	**MVPA+** n = 67	**MVPA-** n = 67	**SED-/MVPA+** n = 33	**SED-/MVPA-** n = 34	**SED+/MVPA+** n = 33	**SED+/MVPA-** n = 34
**Anthropometry**								
Age (year)	13.1 ± 2.3	13.6 ± 1.9	13.0 ± 2.3	13.7 ± 1.9	12.4 ± 2.3	13.6 ± 2.2	13.1 ± 1.8	14.2 ± 1.9
Females (n, %)	33 (49)	32 (47)	33 (49)	32 (47)	15 (45)	17 (50)	16 (48)	18 (52)
Tanner stage	3.7 ± 1.3	3.9 ± 1.3	3.7 ± 1.3	3.9 ± 1.3	3.4 ± 1.5	3.9 ± 1.3	3.7 ± 1.3	4.1 ± 1.2
Height (cm)	161.2 ± 16.2	167.1 ± 9.7	159.3 ± 14.8	168.8 ± 10.4	152.6 ± 14.4	169.4 ± 13.5	165.2 ± 9.3	168.8 ± 9.9
Weight (kg)	87.6 ± 28.8	98.9 ± 19.5**	83.8 ± 25.2	102.7 ± 21.3***	74.3 ± 22.4	100.6 ± 28.8 ##	94.9 ± 20.4 ##	102.9 ± 17.8 ##
BMI ($kg \cdot m^{-2}$)	32.9 ± 5.5	35.6 ± 4.5*	32.6 ± 4.8	36.1 ± 5.0**	31.3 ± 4.6	34.6 ± 5.9	35.3 ± 4.8	36.0 ± 4.3 #
SDS-BMI	3.19 ± 0.51	3.26 ± 0.45	3.14 ± 0.48	3.22 ± 0.48	3.24 ± 0.54	3.14 ± 0.48	3.18 ± 0.48	3.17 ± 0.43
BMI (percentile)	98.8 ± 0.8	99.0 ± 0.7*	98.8 ± 0.8	99.0 ± 0.7**	98.7 ± 0.8	98.8 ± 0.8	98.9 ± 0.7	99.1 ± 0.6
WC (cm)	105.8 ± 15.1	112.3 ± 11.5*	103.7 ± 13.8	114.3 ± 11.7**	99.9 ± 11.9	110.9 ± 12.3	111.6 ± 15.9	113.6 ± 10.8 #
**Accelerometry**								
Sedentary time (min/day)	557 ± 38	723 ± 109***	584 ± 61	696 ± 171***	553 ± 44	562 ± 31	674 ± 50 ### $$$	769 ± 129 ### $$$ †††
LPA (min/day)	533 ± 88	433 ± 103***	494 ± 80	473 ± 111	496 ± 82	570 ± 78	460 ± 59 $$$	408 ± 127 ** $$ †
MPA (min/day)	222 ± 75	149 ± 59***	248 ± 50	124 ± 84***	281 ± 46	165 ± 49 ###	197 ± 37 ### $$	104 ± 35 ### $$$ †††
VPA (min/day)	8 ± 14	4 ± 5*	10 ± 13	2 ± 3***	13 ± 18	4 ± 5 ###	6 ± 5	2 ± 2 ### †††
MVPA (min/day)	231 ± 81	153 ± 6***	258 ± 56	127 ± 86***	294 ± 53	169 ± 51###	203 ± 38 ###$$$	106 ± 36 ### $$ †††
Total PA (min/day)	764 ± 72	586 ± 135***	752 ± 81	599 ± 144***	790 ± 65	739 ± 71	663 ± 68 #	514 ± 143 ### $$$ †††
**Cardiometabolic**								
Systolic BP (mmHg)	116 ± 12	122 ± 11*	116 ± 11	122 ± 11 *	114 ± 10	118 ± 13	121 ± 12	122 ± 9
Diastolic BP (mmHg)	70 ± 9	74 ± 8*	70 ± 8	73 ± 9	68 ± 7	70 ± 9	73 ± 8	74 ± 8
HDL-cholesterol (mmol/L)	1.16 ± 0.24	0.96 ± 0.20**	1.14 ± 0.25	0.98 ± 0.21**	1.23 ± 0.25	1.09 ± 0.22	0.97 ± 0.19 ## $	0.95 ± 0.21 ## $
Triglycerides (mmol/L)	1.09 ± 0.56	1.36 ± 0.58*	1.07 ± 0.53	1.38 ± 0.60*	1.08 ± 0.59	1.10 ± 0.54	1.34 ± 0.55	1.37 ± 0.62
LDL-cholesterol (mmol/L)	2.50 ± 0.71	2.71 ± 0.89	2.51 ± 0.77	2.70 ± 0.84	2.66 ± 0.90	2.36 ± 0.42	2.64 ± 0.79	2.77 ± 0.98
Total cholesterol (mmol/L)	3.88 ± 0.83	3.96 ± 0.96	3.87 ± 0.87	3.96 ± 0.92	4.10 ± 1.02	3.67 ± 0.52	3.88 ± 0.85	4.00 ± 1.07
Fast glucose (mmol/L)	5.39 ± 0.49	5.72 ± 0.64**	5.51 ± 0.60	5.59 ± 0.59	5.37 ± 0.47	5.41 ± 0.50	5.70 ± 0.6 8 # $	5.75 ± 0.6 # $
Fast insulin (mUI/L)	19.24 ± 11.41	28.93 ± 15.19***	20.15 ± 13.18	28.03 ± 14.29**	17.92 ± 9.68	20.48 ± 12.86	28.83 ± 15.0 # $	29.02 ± 13.46 # $
HOMA-IR	4.57 ± 2.94	7.46 ± 4.23***	4.97 ± 3.74	7.06 ± 3.82**	4.20 ± 2.69	4.92 ± 3.15	7.43 ± 4.71 ### $$	7.50 ± 3.78 ### $$

BMI: body mass index; BP: blood pressure; HDL: high-density lipoprotein; HOMA-IR: homeostasis model assessment of insulinresistance; IR: insulinresistance; LDL: low density lipoproprotein; LPA: light physical activity; MetS: metabolic syndrome; MPA: moderate physical activity; MVPA: moderate to vigorous physical activity; PA: physical activity; TG : triglycerides ; VPA: vigorous physical activity

*Different between SED+ vs SED- groups and between MVPA- vs MVPA+ groups: *p<0.05,** p<0.01, ***p<0.001*

Different from SED-MVPA+: # p<0.05 ; ## p<0.01 ; ### p<0.001

Different from SED-MVPA- : $ p< 0.05 ; $$ p<0.01 ; $$$ p<0.001

Different from SED+MVPA+ ; † p < 0.05 ; †† p<0.01 ; ††† p<0.001
